# The impact of joint attention on the sound-induced flash illusions

**DOI:** 10.3758/s13414-021-02347-5

**Published:** 2021-09-24

**Authors:** Lucas Battich, Isabelle Garzorz, Basil Wahn, Ophelia Deroy

**Affiliations:** 1 Graduate School of Systemic Neurosciences, Ludwig-Maximilians-Universität München, Munich, Germany; 2 Faculty of Philosophy, Philosophy of Science and Religious Studies, Ludwig-Maximilians-Universität München, Geschwister-Scholl-Platz 1, 80359 Munich, Germany; 3 Department of Psychology, University of British Columbia, Vancouver, Canada; 4 Department of Psychology, Leibniz Universität Hannover, Hannover, Germany; 5 Munich Center for Neuroscience, Ludwig-Maximilians-Universität München, Munich, Germany; 6 Institute of Philosophy, School of Advanced Study, University of London, London, England

**Keywords:** Joint attention, Multisensory integration, Sound-induced flash illusion

## Abstract

Humans coordinate their focus of attention with others, either by gaze following or prior agreement. Though the effects of joint attention on perceptual and cognitive processing tend to be examined in purely visual environments, they should also show in multisensory settings. According to a prevalent hypothesis, joint attention enhances visual information encoding and processing, over and above individual attention. If two individuals jointly attend to the visual components of an audiovisual event, this should affect the weighing of visual information during multisensory integration. We tested this prediction in this preregistered study, using the well-documented sound-induced flash illusions, where the integration of an incongruent number of visual flashes and auditory beeps results in a single flash being seen as two (fission illusion) and two flashes as one (fusion illusion). Participants were asked to count flashes either alone or together, and expected to be less prone to both fission and fusion illusions when they jointly attended to the visual targets. However, illusions were as frequent when people attended to the flashes alone or with someone else, even though they responded faster during joint attention. Our results reveal the limitations of the theory that joint attention enhances visual processing as it does not affect temporal audiovisual integration.

People devote greater cognitive resources to those features in their environment that are co-attended simultaneously with others (Becchio et al., 2008; Shteynberg, 2015, 2018). Even in the absence of communication, jointly attending to the same object enhances a participant’s mental spatial rotation performance (Böckler et al., 2011) and facilitates information encoding in working memory (Gregory & Jackson, 2017; Kim & Mundy, 2012). A prevalent theoretical hypothesis regarding the functional role of joint attention is therefore that it deepens or enhances the encoding of stimulus information in ways that are not observed when information is individually attended (Mundy, 2016, 2018; see also Becchio et al., 2008; Shteynberg, 2015). This hypothesis would explain why joint attention plays a fundamental role in language acquisition, the development of theory of mind, and the ability to engage in more complex activities with others (Bottema-Beutel, 2016; Carpenter et al., 1998; Mundy & Newell, 2007).

The hypothesis of an ‘encoding enhancement’ also accords with findings on the influence of joint attention on perceptual judgements. Gaze-cueing studies using a covert shift of attention (i.e., a shift without an eye movement), show that another’s gaze behaviour can influence the detection and discrimination of visual stimuli (see Frischen et al., 2007, for a review). For example, participants are slower in judging the number of visual stimuli presented at a given location if an avatar is looking at a different location, rather than the same location (Samson et al., 2010). While most studies use response times as their primary dependent measure, Seow and Fleming (2019) report that participants also had a better perceptual sensitivity (*d'*) for detecting Gabor patches when they were looking at the same location as another bystander.

In everyday situations, however, joint attention takes place in a multisensory setting, where information from different senses has either to be selected and integrated or, on the contrary, separated (Battich et al., 2020). Previous work addressing the multisensory aspects of joint attention in adults focuses predominantly on spatial judgments. Soto-Faraco et al. (2005) and De Jong and Dijkerman (2019) both report that people are better at detecting and discriminating tactile stimuli on a body location when it is attended by another observer, represented by eye gaze cues. Extending these results, Nuku and Bekkering (2010) show that gaze cues from a virtual partner also influence spatial auditory judgements, but only if the partner can also hear the sounds.

So far, then, findings in the multisensory domain support the hypothesis that visually attending to a given location with someone will enhance the encoding and processing of any sensory stimuli presented in that location, compared with solo attention (Mundy, 2016, 2018). Joint attention, however, is not directed only toward objects and their locations, but to multisensory events extended through time. Specifically, it is unknown to what extent joint attention impacts how multisensory events are integrated. Since the ‘encoding enhancement’ hypothesis can account for a range of joint attentional effects on perceptual judgments, it is necessary to confirm systematically its explanatory extent, especially in the context of multisensory integration in the temporal domain. If we are better at perceiving visual stimuli presented in a jointly attended location, will we also be better at counting visual events relative to sounds presented closely in time, when we attend to them with someone else? This is the hypothesis that we test in this study. Specifically, we examine whether jointly attending to visual stimuli would also result in enhancing their processing, and make people less influenced by distracting sounds. Target enhancement and reduction of distractors are often considered two sides of the same coin, yet mechanistic differences provide reasons to regard them as possibly distinct (Chelazzi et al., 2019; Noonan et al., 2016; van Moorselaar & Slagter, 2020).

Moving the joint attention paradigm to the temporal domain makes it more challenging to ask people to both track where another person (or an avatar) is gazing, and when the visual target appears. Tracking where someone is attending is also not necessary for joint attention, which involves representing that the two co-attenders attend to the same perceptual target (Carpenter et al., 1998; Mundy, 2018; Siposova & Carpenter, 2019; Tomasello, 1995). If two or more agents know or infer that they are attending to the same object, they still engage in joint attention even though they are not closely monitoring each other’s gaze (Elekes & Király, 2021).

In this preregistered study (preregistration available at https://osf.io/v5gjp), we manipulated joint attention by letting two participants sit side-by-side and letting them know that they were focusing their attention on the same visual targets of a visual task (joint attention) or one of them performed a different task, not looking at the visual targets (social control). Each person also conducted the visual task alone. In each case, participants were presented with variants of the sound-induced flash illusions, where a single flash accompanied by two auditory beeps are prone to induce an illusory visual percept of two flashes (fission illusion), and where two flashes accompanied by a single auditory beep are prone to be perceived as a single flash (fusion illusion; Andersen et al., 2004; Shams et al., 2000). According to the modality appropriateness hypothesis, the rationale behind the illusions is that the auditory signal dominates over the visual signal in tasks requiring temporal precision, altering the integrated percept (Andersen et al., 2004; Shams et al., 2002). The sound-induced flash illusions are reliably used to investigate the multisensory integration of temporally aligned stimuli (Hirst et al., 2020; Keil, 2020), providing an effective tool to test whether joint attention modulates multisensory integration. What is more, by keeping the spatial relation constant between modal cues and their spatial locations, a sound-induced flash illusions paradigm is ideal to study joint attention as it does not require participants to shift their attention (e.g., to someone’s eye-gaze direction).

While the specific interactions between attention and multisensory processes are a matter of ongoing debate, mounting evidence suggests that multisensory integration can be modulated by attentional control (for reviews, see Choi et al., 2018; Macaluso et al., 2016; Talsma et al., 2010). Several studies report possible cognitive influences on the sound-induced flash illusions (for reviews see Hirst et al., 2020; Keil, 2020), yet earlier studies only investigated manipulations of the individual’s attentional focus. For instance, Andersen et al. (2004) found that the integration of audiovisual information during both fission and fusion illusions was not automatic, but varied depending on whether participants were asked to count beeps or flashes. The illusions seem susceptible to differences in attentional control, an interpretation supported by findings that the fission illusion is modulated by selective spatial attention (Mishra et al., 2010; Odegaard et al., 2016). The fission illusion is also modulated by cognitive load (Michail & Keil, 2018) and top-down expectations about the proportion of illusion-inducing trials (Wang et al., 2019).

However, as current functional models of joint attention suggest that sharing the locus of attention with another person will enhance information processing in ways that solo attention does not (Battich et al., 2020; Mundy, 2018), it is important to investigate how a joint attention manipulation may affect multisensory integration, besides known attentional effects in individual settings. Investigating such manipulation is relevant also for experimenters as they may need to reconsider the possible effects of being within a participant’s view while testing the sound-induced flash illusions (Hirst et al., 2020). It is known, for example, that the presence of another person may increase motivation or arousal, leading to social facilitation effects, even when not attending to the same target (Belletier et al., 2019; Steinmetz & Pfattheicher, 2017). The present study thus informs one of the outstanding questions in multisensory research: whether and how social factors affect multisensory processes.

Using the sound-induced flash illusions also affords comparisons with recent reports that dividing attentional labour increases susceptibility to the fission illusion (Wahn et al., 2020). In their study, the tasks were divided across sensory modalities: the participant was asked to count the number of flashes, either with a confederate counting the number of beeps, or alone. Participants were more susceptible to fission illusions in the social compared with the individual condition. However, this effect was no longer found when a divider was placed between the participant and confederate, suggesting that common visual access is critical. Taken together with previous studies (Heed et al., 2010; Wahn et al., 2017) where participants were better able to ignore distracting *visual* stimuli in multisensory tasks, the authors suggest that the presence of another person may act as a visual distractor so that visual information presented on the screen is attended to a lesser extent. Depending on the task, this effect would improve performance when the participant had to ignore visual information during a tactile (Heed et al., 2010) or auditory (Wahn et al., 2017) localization task, and worsen performance when participants had to count visual targets and ignore auditory distractors (Wahn et al., 2020). There is no previous indication, to our knowledge, of whether a similar or opposite effect would be observed in a joint attention manipulation, where both participants in a pair are required to attend and respond to the same modal target. Importantly, if physical presence and common visual access are sufficient, participants will be more prone to illusions when attending to flashes with others rather than alone. In contrast, the hypothesis that joint attention enhances the encoding of visual information relative to the auditory distractors predicts the opposite.

If engaging in joint attention enhances processing of a jointly attended visual target (Becchio et al., 2008; Mundy, 2016, 2018; Shteynberg, 2015, 2018), we predict a shift in the relative weighting of visual and auditory information, so that the strength of the sound-induced illusions will be reduced during joint attention compared with performing the task alone (see Fig. 1d). To control for the mere effect of co-presence, we added a social condition where one participant was performing the flash counting task, and the other was engaged in another task not looking at the screen. We predicted that participants’ performance would not be different when performing the flash counting task alone or in the mere presence of someone else, engaged in a different task.

**Fig. 1:**
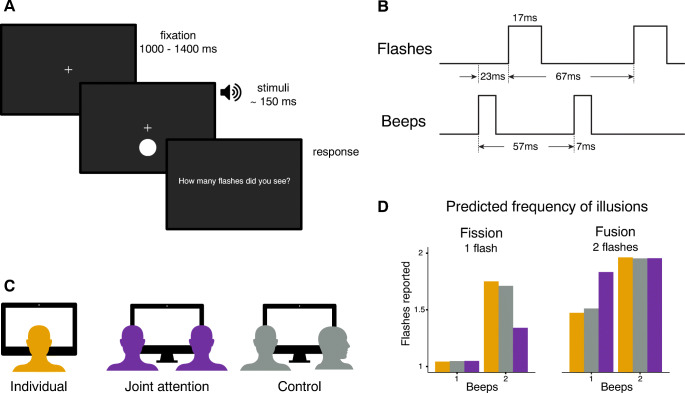
**1** Single trial procedure. **b** Temporal order of stimuli (when two flashes and two beeps are presented). **c** Experimental set-up for each social condition. **d** Predicted frequency of flashes reported when one flash (fission) and two flashes (fusion) are presented, according to the joint attention ‘encoding enhancement’ hypothesis

Finally, while we predicted that both fission and fusion illusions would diminish during joint attention, we had no specific predictions regarding possible differences between them. To date, there is no reliable previous indication that our social manipulation should affect the illusions differently, as previous studies on the cognitive influences on the illusions have been predominantly focused on the fission illusion (Michail & Keil, 2018; Mishra et al., 2010; Odegaard et al., 2016). Nonetheless, known neural (Mishra et al., 2008; Innes-Brown et al., 2013; Watkins et al., 2007) and behavioural differences between the fission and fusion illusions suggest that we should not treat them as necessarily identical either (Hirst et al., 2020). Susceptibility to the fission illusion, but not the fusion illusion, varies with age (DeLoss & Andersen, 2015; McGovern et al., 2014) and with emotionally charged stimuli (Takeshima, 2020). Importantly, there is preliminary evidence that cognitive expectations (Wang et al., 2019) decrease the occurrence of fission, but not fusion illusions. Given these potential differences in the mechanisms underlying the fission and fusion illusions, our study tests the influence of joint attention for both illusions.

## Material and methods

### Participants

Given current literature on possible social effects on the sound-induced flash illusion, our estimate of a Cohen’s *d* effect size is 0.41 (Wahn et al., 2020). We used the software G*Power (Faul et al., 2007) to conduct a power analysis, to obtain .80 power to detect Cohen’s *d* effect size of 0.415 for a two-tailed paired *t* test, at the standard .05 alpha error probability. Our target sample size was 48 participants. Due to the possibility of some participants not meeting the inclusion criteria, we recruited 52 volunteers (29 female, one undisclosed gender, *M* = 27.96 years, *SD* = 5.9 years) to participate in the study. Participants chose to receive either 9 EUR or course credits as compensation for their participation. All participants had normal or corrected-to-normal vision and hearing, and were right-handed, with mean handedness score *M* = 95.26, *SD* = 15.18, as measured by the shortened Edinburgh Handedness Inventory (Oldfield, 1971; Veale, 2014).

The study was conducted in accordance with the Declaration of Helsinki and approved by the ethics committee of the University of London (approval ref. SASREC_1819_313A). All participants gave written informed consent before their participation.

### Materials

Pairs of participants sat next to each other in front of the same computer screen (model Asus VG248QE 24 inches, of 1,920 × 1,080 pixels resolution, and 60 Hz refresh rate), and at a fixed viewing distance (60 cm) from the screen. Their heads were aligned to the outer edges of the screen (width 53 cm), so that when looking straight ahead they see the screen outer edge. Two speakers (model Logitech Z200) were set adjacent to each side of the screen so that the speaker’s middle was levelled with the lower edge of the screen.

A fixation cross was presented for an interval that varied randomly between 1,000 and 1,400 ms, followed by the visual and auditory stimuli (see Fig. 1a). The visual stimulus consisted of a uniform white disc (radius of 2° of visual field, positioned 5° below the fixation cross), flashed for 17 ms, on a black computer screen. The auditory stimulus consisted of a sine-wave beep of 7-ms duration with 3.5 kHz frequency. Stimulus onset asynchrony (SOA) for consecutive stimuli was 57 ms for sound beeps, and 67 ms for visual flashes. The first beep was presented always 23 ms prior to the first flash (see Fig. 1b).

### Procedure

In each trial, either one or two flashes were presented, accompanied by either one or two beeps, giving four types of trials (1F1B, 1F2B, 2F1B, 2F2B). Each of the four types of trials was presented 30 times. The 120 trials were fully randomized and presented in four blocks with approximately 10 seconds rest between blocks. Participants were asked to judge how many visual flashes they saw and respond as soon and correctly as possible, by clicking the left or right buttons of a computer mouse allocated to each participant, to report one or two flashes, respectively. Both participants were given the same instructions simultaneously and knew that they were performing the same task.

Participants performed the full set of 120 trials three times, one per social condition: individually, jointly, and during a co-presence control (see Fig. 1c). In the individual condition, participants sat alone to perform the task, in the same seat that they occupy during the joint attention and co-presence control conditions (i.e., a given participant always had the same seat); the second participant waited in a separate testing room. During the joint attention condition, both participants were instructed to attend to the visual stimuli and perform the task concurrently. Each participant still provided their answer individually. Participants were informed that their task was exactly the same as when performing alone. No feedback was provided, neither on their own nor the other’s responses and results. In the co-presence control condition, participants sat side by side as in the joint attention condition but oriented in opposite directions. One participant performed the flash-counting task, while the second participant performed an unrelated drawing task on paper. Then participants switched roles. During the 120 trials of each condition, the experimenter waited outside the testing room, out of sight from both participants. Participants were instructed to avoid talking to each other during the flash-counting task. Due to the short duration of each trial and the demanding nature of the flash-counting task, verbal communication is also very difficult to achieve. After 120 trials were completed, participants saw a text on the screen requesting that the experimenter should be contacted. The experimenter then made the necessary setup adjustment depending on the next social condition, instructing each participant on their assigned role (e.g., to perform the flash-counting task, wait in an adjacent room, or perform an unrelated drawing task). The order of social conditions was counterbalanced across participants. In most cases, the session took approximately 45 minutes. The experiment was programmed using Python (Version 3.6.8) and the PsychoPy library (Version 3.2.3; Peirce, 2007; Peirce et al., 2019).

### Data analysis plan

To analyze the effect of joint attention on the strength of the illusions, we preregistered to conduct a 2 × 3 repeated-measures analysis of variance (ANOVA) for the mean responses with beeps (one, two beeps) and social condition (individual, joint attention, control) as within-subject factors, separately for the fission (one flash trials) and fusion (two flashes trials) illusions.

We also preregistered and planned two paired *t*-test comparisons over the interaction effects between beeps and social conditions on the number of flashes perceived. First, to test whether joint attention reduced the illusions, we contrasted the effect of beeps on the number of flashes reported across the individual and joint attention condition. Second, to test whether the mere presence of another participant affects the frequency of the illusions, we contrasted the effect of beeps on the number of flashes reported across the individual and control condition. We performed these planned comparisons regardless of whether the omnibus interaction was significant (Abelson & Prentice, 1997; Schad et al., 2020).

Since the assumption of normality in the parametric models for the number of flashes perceived (ANOVAs and *t* tests) was violated (Shapiro–Wilk tests performed on the data were significant, all *p*s < .001), we conducted permutation-based ANOVAs separately for each illusion (one flash and two flashes trials). We then performed the two planned pairwise comparisons with permutation-based *t* tests, for each illusion. Though all comparisons were planned, we report *p* values corrected using the Bonferroni correction.

We excluded three participants from the sample due to low performance (greater than or equal to 35% incorrect responses) on either or both of congruent trials combinations (equal number of flashes and beeps presented), aggregated across social conditions, probably due to lack of motivation or task compliance. Only single trials with reaction times between 100 ms and 3,000 ms were included in the analyses. We thus excluded 0.5% of trials (92 trials) spread over 24 participants from further analyses.

To follow upon performance analyses, we preregistered to conduct exploratory analyses on any possible effects over reaction times across the different experimental manipulations. To examine performance measures that better account for possible dissociations in sensitivity and criterion biases, we also preregistered to analyze possible effects on signal detection measures in the ability to discriminate between one and two flashes, during two-beep trials (coded as Fission), and one-beep trials (coded as Fusion). Note that the underlying data for coding each illusion differs from the performance analyses above.

Witt et al. (2015, 2016) suggest that the sound-induced flash illusions should be reflected primarily in the criterion measure as indicative of perceptual processes. Theoretically, the number of beeps biases visual perception to detect the same number of flashes rather than making visual perception less sensitive per se. Knotts and Shams (2016) suggest that both *d'* and c may reflect perceptual aspects associated with the illusion. An analysis of sensitivity and criterion can therefore provide nuanced measures for testing the impact of social conditions on the illusions. It would be a mistake, however, to interpret the criterion bias as a decision bias, response-based bias, or memory bias. Witt et al. (2015, 2016) show that the sound-induced flash illusions are predominantly manifested in the criterion measure *c*, but we are not able to distinguish by SDT techniques alone if this bias is perceptual or decisional. The single flash stimulus was treated as the target, so that a correct response of one flash when one flash was presented was counted as a hit, and an incorrect response of one flash when two flashes were presented was counted as a false alarm. Sensitivity was defined as *d* ′  = *z*(*H*) − *z*(*FA*), and criterion bias was defined as *c* =  − .5(*z*(*H*) + *z*(*FA*)), where *z* is the inverse of the cumulative normal. Hit and false-alarm rates of 0 and 1 were corrected to (2*N*)^−1^ and 1 − (2*N*)^−1^, respectively, where *N* is the number of trials on which the rate is based (Macmillan & Creelman, 2005).

For each illusion, we performed one-way repeated measures ANOVAs of *d'* and *c*, dependent on social condition as a within-subject factor. As in our performance analysis, we then conducted two planned pairwise comparisons (individual vs. joint attention, and individual vs. control), reported with Bonferroni corrected *p* values.

## Results

### Fission illusion

#### Number of flashes perceived

**Fig. 2:**
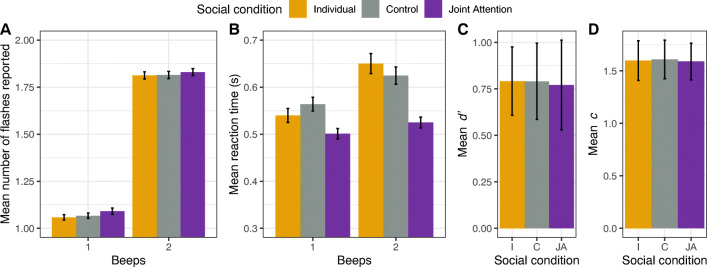
Fission illusion results. **a** Mean number of flashes reported. **b** Mean reaction times in 1F1B and 1F2B trials across conditions. **c**–**d** Signal detection measures of sensitivity (*d'*) and bias (*c*) in the ability to discriminate between one and two flashes during 1F2B and 2F2B trials. Error bars show within-subjects adjusted 95% confidence intervals (Cousineau, 2005; Morey, 2008)

Figure 2a shows the overall mean of each participant’s mean responses in trials where a single flash was presented. Trials with two beeps display a strong increase in the average number of flashes reported. Table 1 shows the mean number of flashes reported and reaction times for all conditions. To test the effect of the social manipulations, we subjected the number of flashes perceived in one-flash trials to a permutation-based repeated-measures ANOVA (Kherad-Pajouh & Renaud, 2015) with beeps (1, 2 beeps) and social condition (individual, joint attention, control) as within-subject factors. We found a significant main effect of beeps, *F*(1, 48) = 521.78, *p* < .001, ${\eta}_g^2$ = .8. When one flash was presented, the number of beeps affected the number of flashes reported, showing that this audiovisual manipulation successfully induced a fission illusion. However, we did not find a significant main effect of social condition, *F*(2, 96) = 2.41, *p* = .09, ${\eta}_g^2$ = .004, nor an interaction effect, *F*(2, 96) = 0.16, *p* = .84, ${\eta}_g^2$ < .001.

**Table 1:** Mean number of flashes reported and mean response times (RTs) for each stimulus type across social conditions

	Individual		Control		Joint attention	
Stimulus	Flashes reported	RTs (s)	Flashes reported	RTs (s)	Flashes reported	RTs (s)
1F1B	1.06 (0.23)	0.54 (0.29)	1.07 (0.25)	0.56 (0.29)	1.09 (0.29)	0.5 (0.2)
1F2B	1.81 (0.39)	0.65 (0.44)	1.81 (0.39)	0.62 (0.38)	1.83 (0.38)	0.52 (0.22)
2F1B	1.38 (0.48)	0.65 (0.4)	1.39 (0.49)	0.65 (0.4)	1.41 (0.49)	0.54 (0.21)
2F2B	1.96 (0.19)	0.58 (0.37)	1.95 (0.21)	0.55 (0.33)	1.95 (0.21)	0.49 (0.2)

*Note.* Standard deviations are included in parentheses.

Although the interaction was not significant, we performed the preregistered planned permutation-based paired *t* test on the effect of beeps on the number of flashes reported (the difference in responses across one- and two-beep trials) between the individual and joint attention conditions. Contrary to our hypothesis, we found no significant difference, *t*(48) = −0.45, *corrected p* = 1, Cohen’s *d* = 0.06. As these results suggest that engaging in joint attention does not affect susceptibility to the fission illusion, we also computed Bayes factors (BF) for this effect to assess relative likelihoods of the null (H0) and alternative (H1) hypotheses (we note that Bayes factor analyses were not included in our preregistration). BF = 1 indicates equal support for H1 and H0, while BFs between 1 and 3, 3 and 10, and >10 indicate anecdotal, moderate, and strong support for H1, respectively, and BFs between .33 and 1, .1 and .33, and <.1 indicate anecdotal, moderate, and strong support for H0, respectively (Aczel et al., 2017). We found a Bayes factor of .17, indicating that our data give moderate support for the null hypothesis (it is 5.88 more likely under the null than under the alternative hypothesis).

As expected, we found no significant differences in the pairwise comparisons between individual and control conditions on the difference in responses across one- and two-beep trials, *t*(48) = −0.20, *corrected p* = 1, Cohen’s *d* = 0.03. A computed Bayes factor of .16 indicates moderate support for the null hypothesis, so that our data are 6.3 times more likely under the null than under the alternative hypothesis. These results suggest that participants were susceptible to the fission illusion, but this susceptibility did not differ between social conditions.

#### Reaction times

Figure 2b shows the overall mean of each participant’s mean reaction times in trials where a single flash was presented. To test whether the observed difference in latencies across social conditions was significant, we subjected the reaction times to a permutation-based repeated measures ANOVA with beeps (1, 2 beeps) and social condition (individual, joint attention, control) as within-subject factors. We found a significant main effect of beeps, *F*(1, 48) = 9.69, *p* < .01, ${\eta}_g^2$ = .03, and a significant effect of social condition, *F*(2, 96) = 10.45, *p* < .001, ${\eta}_g^2$ = .04. The interaction effect was small though significant with *F*(2, 96) = 7.78, *p* < .001, ${\eta}_g^2$ = .009. We followed this interaction effect with three permutation-based pairwise comparisons, comparing the difference between congruent (one flash, one beep) and incongruent (one flash, two beeps) presentations between social conditions. We found that this congruent-incongruent difference was significantly reduced in the joint attention condition compared with the individual condition (*t*(48) = -3.43, *corrected p* = .006, Cohen’s *d* = 0.48), and did not significantly differ between individual and control conditions, *t*(48) = −2.27, *corrected p* = .078, Cohen’s *d* = 0.32, nor between control and joint attention conditions, *t*(48) = −2.01, *corrected p* = .19, Cohen’s *d* = 0.28. Our results indicate that, for comparable performance, the response speed difference between congruent and incongruent trials observed during the individual condition disappeared in the joint attention condition.

#### Signal detection measures

Signal detection theory analysis indicated that sensitivity and criterion bias did not visibly differ across social conditions (see Fig. 2c–d, respectively). One-way repeated ANOVAs showed neither a significant effect of social condition on sensitivity *d'*, *F*(2, 96) = 0.03, *p* = .96, nor on criterion *c*, *F*(2, 96) = 0.03, *p* = .96. Similarly, our pre-planned pairwise comparisons did not reveal significant differences for *d'* and *c* (see Table 2).

**Table 2:** Pairwise compassions of signal detection measures across social conditions for the fission illusion

Measure	Comparison	*t*	*df*	95% CI	Cohen’s *d*	Corrected *p*
Sensitivity *d'*	Individual vs. joint attention	0.22	48	[−0.17, 0.21]	0.03	1
	Individual vs. control	0.01	48	[−0.16, 0.16]	0.00	1
Criterion *c*	Individual vs. joint attention	0.11	48	[−0.15, 0.17]	0.02	1
	Individual vs. control	−0.13	48	[−0.17, 0.15]	0.02	1

*Note*. CI = confidence interval; Bonferroni corrected *p* values.

### Fusion illusion

#### Number of flashes perceived

**Fig. 3:**
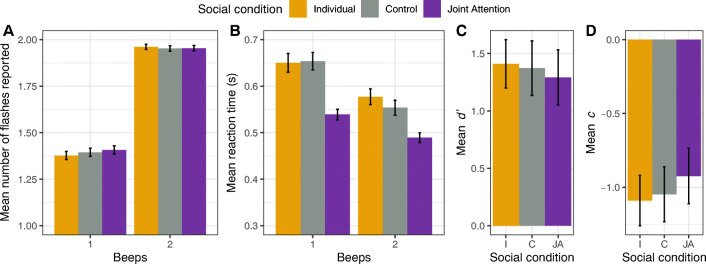
Fusion illusion results. **a** Mean number of flashes reported. **b** Mean reaction times in 2F1B and 2F2B trials across conditions. **c–d** Signal detection measures of sensitivity (*d'*) and bias (*c*) in the ability to discriminate between one and two flashes during 1F2B and 2F2B trials. Error bars show within-subjects adjusted 95% confidence intervals (Cousineau, 2005; Morey, 2008)

We performed the same analyses to assess the effect on the fusion illusion, where two flashes were presented, as we did for the fission illusion. Figure 3a shows the overall mean of each participant’s mean responses in two-flashes trials. Trials with one beep showed a decrease in the average number of flashes reported, so that when two flashes and one beep were presented concurrently, participants tended toward reporting one flash. Table 1 shows the mean number of flashes reported and reaction times for all conditions. We subjected the number of flashes reported in two-flashes trials to a permutation-based repeated-measures ANOVA with beeps (1, 2 beeps) and social condition (individual, joint attention, control) as within-subject factors. We found a significant main effect of beeps on the mean flashes reported, *F*(1, 48) = 144.59, *p* < .001, ${\eta}_g^2$ = .55, showing that participants were susceptible to the fusion illusion. However, we did not find a significant main effect of social condition, *F*(2, 96) = 0.26, *p* = .78, ${\eta}_g^2$ < .001, or interaction effect, *F*(2, 96) = 0.9, *p* = .4, ${\eta}_g^2$ < .001.

Although the interaction effect was not significant, we performed two planned comparisons as pre-registered. To test the hypothesis that the illusion diminishes during joint attention as compared with individual performance, we computed the difference in responses across one- and two-beep trials, and then performed a permutation-based paired *t* test between the individual and joint attention conditions. Contrary to our hypothesis, we found no significant differences, *t*(48) = 1.49, *corrected p* = .22, Cohen’s *d* = 0.21. These results suggest that engaging in joint attention does not affect susceptibility to the fusion illusion. To assess the relative likelihoods of the null and alternative hypotheses, we computed Bayes factors for this comparison and found that with a Bayes factor of .43, our data provides anecdotal support in favour of the null hypothesis, so that the data is 2.27 more likely under the null than the alternative hypothesis. As expected, we found no significant differences in the pairwise comparisons between individual and control conditions on the difference in responses across one- and two-beep trials, *t*(48) = −0.83, *corrected p* = .85, Cohen’s *d* = 0.12. Our computed Bayes factor of .21 indicates moderate support for the null hypothesis, so that our data are 4.64 times more likely under the null than the alternative hypothesis.

These results suggest that participants were susceptible to the fusion illusion, yet this susceptibility did not differ between social conditions. Unlike the results for the fission illusion, however, our data provides only anecdotal support for the null hypothesis that there are no differences between individual and joint attention conditions.

#### Reaction times

Figure 3b shows the overall mean of each participant’s mean reaction times in trials where two flashes were presented. We subjected the reaction times to a permutation-based repeated-measures ANOVA with beeps (1, 2 beeps) and social condition (individual, joint attention, control) as within-subject factors. We found a significant main effect of beeps, *F*(1, 48) = 32.64, *p* < .001, ${\eta}_g^2$ = .03, and a significant effect of social condition, *F*(2, 96) = 11.62, *p* < .001, ${\eta}_g^2$ = .05; yet the interaction effect was not significant, *F*(2, 96) = 2.23, *p* = .11, ${\eta}_g^2$ = .002. Bonferroni post hoc tests revealed significantly lower response times in the joint attention condition compared with both individual (*p* < .001) and control (*p* < .001) conditions, but no significant difference between the individual and the control conditions (*p* = .99).

Mirroring our performance analyses, we then performed two pre-planned pairwise comparisons with permutation-based *t* tests on the computed difference in reaction times between one and two beeps for each social condition. We found no significant differences between individual and joint attention conditions, *t*(48) = −1.31, *corrected p* = .36, Cohen’s *d* = 0.18, and neither between individual and control conditions, *t*(48) = 0.75, *corrected p* = .95, Cohen’s *d* = 0.11.

These results indicate that participants were faster during congruent (two flashes, two beeps) than incongruent (two flashes, one beep) stimuli, and faster in the joint attention condition compared with the individual or control condition, for comparable performance on the flash-counting task.

#### Signal detection measures

Signal detection theory analysis indicated that sensitivity and criterion bias did not visibly differ across social conditions (see Fig. 3c–d, respectively). One-way repeated ANOVAs showed no significant effect of social condition on sensitivity *d'*, *F*(2, 96) = 0.53, *p* = .58, and neither on criterion *c*, *F*(2, 96) = 2.75, *p* = .07. As in our performance analyses, we performed two pre-planned pairwise comparisons for differences in *d'* and *c*, between individual and joint attention conditions, and between individual and control conditions (see Table 3). As shown in this table, we found a significant difference in the decision criterion *c* between joint attention (*M* = −0.92, *SD* = 0.85) and individual (*M* = −1.09, *SD* = 0.77) conditions, suggesting that participants were less biased toward the auditory distractor during joint attention, as compared with performing the task individually. This reduced bias was not observed between the individual and control conditions.

**Table 3:** Pairwise comparisons of signal detection measures across social conditions for the fusion illusion

Measure	Comparison	*t*	*df*	95% CI	Cohen’s *d*	Corrected *p*
Sensitivity *d'*	Individual vs. joint attention	1.02	48	[−0.11, 0.35]	0.15	0.63
	Individual vs. control	0.34	48	[−0.18, 0.25]	0.05	1.00
Criterion *c*	Individual vs. joint attention	−2.34	48	[−0.31, −0.02]	0.33	0.04
	Individual vs. control	−0.58	48	[−0.19, 0.1]	0.08	1.00

*Note*. CI = confidence interval; Bonferroni corrected *p* values.

## Discussion

In this study, we investigated whether the hypothesis that joint attention can boost relative processing of co-attended sensory stimuli compared with solo attention (Becchio et al., 2008; Mundy, 2016, 2018; Shteynberg, 2015, 2018) extends to temporal multisensory integration. Specifically, we tested whether engaging in joint attention could reduce temporal audiovisual illusions by enhancing the processing of the jointly attended modality and/or reducing the distraction to the non-attended modality.

Previous work examined the impact of joint attention on stimuli in the tactile or auditory modality with artificial gaze cues displayed on a computer screen (De Jong & Dijkerman, 2019; Nuku & Bekkering, 2010; Soto-Faraco et al., 2005). Here, we investigated the impact of joint attention on *audiovisual* stimuli and manipulated joint attention by having two participants concurrently know that they are attending to the same visual target. Using the sound-induced flash illusions, participants counted visual flashes in three social conditions: alone, in pairs sitting in proximity, and with another participant sitting in proximity but with their attention engaged in a different task. In all social conditions, participants could not ignore the sounds simultaneously presented, and were susceptible to both seeing more (i.e., the fission illusion) or fewer flashes (i.e., the fusion illusion) than actually presented. With these findings, we replicate previous studies that focused on individual performance (Andersen et al., 2004; Keil, 2020; Shams et al., 2002). Following the hypothesis that joint attention enhances relative information encoding and processing of the co-attended stimuli relative to distractors, we predicted that when participants jointly attend and respond to the same visual target stimuli, the sound-induced flash illusions will be reduced. However, people did not perform better or worse across the different social conditions, in both fission and fusion illusions. These findings suggest that the temporal integration of audiovisual stimuli, as measured by the number of flashes reported when presented with incongruent beeps, is robust across all social conditions tested.

Regarding reaction times, people performed faster on the joint attention condition compared with the other social conditions across all stimuli combinations. Despite no incentive to compete, and the fact that they could not track the other’s responses or results, people could have still been under a form of social comparison. However, social comparison is most pronounced when people know each other or when the task is personally relevant (Garcia et al., 2013), which did not apply to this study. We suggest that the effect on response times may be due to a social impact on motivation or arousal—a social facilitation effect (Belletier et al., 2019; Steinmetz & Pfattheicher, 2017). These faster responses, however, did also not result in more incorrect responses. That is, as reported above, the accuracy of reported flashes did not differ.

Using signal detection measures, we found that people’s criterion bias was less affected by the auditory beeps for the fusion illusion (i.e., their bias decreased) when engaged in joint attention as contrasted with the individual condition. Such an effect was not observed when comparing the individual and the co-presence control conditions. Interestingly, we only observed a bias reduction on the fusion illusion, while this was not the case for the fission illusion, suggesting that a joint attention manipulation only affects the bias for the fusion but not for the fission illusion. In line with earlier work (Mishra et al., 2008; Watkins et al., 2007; see Hirst et al., 2020, for a review), these findings suggest that the fusion and fission illusions may be mediated by different mechanisms and are thus susceptible to different experimental manipulations.

Recent studies (Tremblay & Nguyen, 2010; Welsh et al., 2020) that examined how performing or observing someone’s actions affects the fusion illusion may help explain our present fusion illusion effects. In particular, Tremblay and Nguyen (2010) found that the fusion illusion is reduced when participants start a goal-directed reaching movement 50 to 100 ms before the audiovisual stimuli are shown. One likely explanation is that during the earlier stages of a goal-directed movement there is a shift in the relative weighting of sensory information towards vision (Kennedy et al., 2015; Manson et al., 2018). In addition, Welsh et al. (2020) report that the fusion illusion is similarly attenuated when participants observe someone else perform the movement, suggesting that participants simulate the performance of the observed action, and thus experience a similar impact on multisensory processing during both action observation and execution. While in our study participants did not engage in any visible motor actions while performing the flash-counting task, one possible interpretation for the reduced bias during the fusion illusion is that the presence of a co-actor engaging in the same task and directing their attention to the same visual target could already (at least minimally) engage the same mechanisms behind the reduction of the fusion illusion during action execution.

One further proviso is needed to interpret this shift in bias. Witt et al. (2015, 2016) show that a change in *c* does not necessarily reflect a change in non-perceptual response bias or decision bias, and that the strength of the sound-induced flash illusions should be reflected primarily in the criterion measure. Theoretically, the number of beeps bias perception to detect the same number of flashes (Witt et al., 2015). Knotts and Shams (2016) suggest that both *d'* and *c* may indicate perceptual processes associated with the illusions. Although we cannot straightforwardly determine whether the bias is either purely perceptual or response based (Witt et al., 2015, 2016), our results indicate that attending to the flashes together with another participant reduces the bias introduced by the sound distractors in the fusion illusion (i.e., discriminating between one and two flashes, when one beep is presented). However, there are no significant social effects in how the mean number of flashes reported differs between one-beep and two-beep trials, when two flashes are presented.

While the present study investigated the impact of joint attention on the sound-induced flash illusion, an earlier study found that a division of labour manipulation, where the participant reported on the number of flashes while a confederate simultaneously reported on the number of beeps, induced a stronger fission illusion compared with performing the task alone (Wahn et al., 2020). The authors suggest that in their social manipulation, the participant’s visual attention was divided between the visual flash-counting task and attending to the co-actor, which in turn increased the influence of the auditory stimuli and thus the number of perceived fission illusions. Since participants in a pair performed different tasks, participants likely showed a tendency to co-represent the other’s task and monitor the other’s performance (Wahn et al., 2020).

For our joint attention manipulation, in contrast, it may not be necessary to co-represent the other person’s task, nor monitor their performance, since the other person attended to the same target and had the same task. Given these differences, the participants’ visual attention was likely not divided in the present study. This interpretation is in line with evidence showing that performing a task together reduces interference in unisensory Stroop-like tasks only when labour is divided, but not when it is shared (Sellaro et al., 2018). In their study, participants had to identify pictures while ignoring distractor words shown concurrently, which induces a semantic interference effect. The interference effect disappeared in the joint task where participants believed that the co-actor was reading the distractor words (different target), but not in the joint task where the co-actor was thought to name the colour of the pictures (same target; Sellaro et al., 2018). Taken together with these studies, the results of the present study indicate that when the participant knows that another actor is taking care of potentially distracting stimuli, a division of labour can be established which affects the participant’s performance. But this effect disappears when both participants are attending and responding to the same target stimulus. In short, multisensory integration of temporal stimuli is affected by a division of labour manipulation but not by a joint attention manipulation.

In the present study, we operationalize joint attention as the situation in which two individuals focus their perceptual attention on the same modal target, and both know together that they are so sharing their attention (Siposova & Carpenter, 2019; Tomasello, 1995). This minimal manipulation is sufficient to induce interferences in the case of joint action (Schmitz et al., 2017). Outside the laboratory, however, joint attention comes in varying degrees, depending on how much co-attenders share between them (Siposova & Carpenter, 2019). Future studies could explore whether factors that elicit a stronger feeling of jointness affect multisensory processing. For instance, the feeling of jointness could be enhanced by reciprocal communicative interaction between co-attenders, sharing emotions (e.g., smiling), sharing object-directed action (e.g., joint intentional goals), familiarity, or previous relationship between the individuals (e.g., family members, friends, partners). The sense of jointness between participants could also depend on the pay-off structure of the task and the required coordination between them. For example, in the absence of a shared goal, an individual can assign little value in co-representing the other’s performance, even though they are engaging in joint attention. In situations where both co-attenders share the same goal, so that they receive greater rewards when their individual performances are aligned, an individual may thus benefit from co-representing the other’s performance and, in turn, their own perceptual processing of the jointly attended target could be thus greatly affected. Future studies could test this proposal, and address the role of different pay-off structures on an individual’s multisensory processing during joint attentional tasks.

Finally, our results shore up the limitations of the view that joint attention enhances stimulus information encoding and processing (Becchio et al., 2008; Mundy, 2018; Shteynberg, 2015). While this view explains the effect of joint attention in facilitating mental spatial rotation performance (Böckler et al., 2011), working memory (Gregory & Jackson, 2017; Kim & Mundy, 2012), and enhancing spatial crossmodal attention (De Jong & Dijkerman, 2019; Nuku & Bekkering, 2010), it cannot be straightforwardly applied to the integration of temporal multisensory events. This study provides grounds for future work in comparing the effects of joint attention across temporal and spatial multisensory processes, and map the limitations of the view that joint attention results in greater processing resources to those features of the environment that are co-attended simultaneously.
